# Risk factors of acute respiratory infections among under five children attending public hospitals in southern Tigray, Ethiopia, 2016/2017

**DOI:** 10.1186/s12887-019-1767-1

**Published:** 2019-10-25

**Authors:** Sielu Alemayehu, Kalayou Kidanu, Tensay Kahsay, Mekuria Kassa

**Affiliations:** Mekelle University, College of Health Sciences, School of Nursing, P.O.B: 1817 Mekelle, Tigray Ethiopia

**Keywords:** Children under 5 years, Acute respiratory infections, Risk factors

## Abstract

**Background:**

Acute Respiratory infection accounts for 94,037000 disability adjusted life years and 1.9 million deaths worldwide. Acute respiratory infections is the most common causes of under-five illness and mortality. The under five children gets three to six episodes of acute respiratory infections annually regardless of where they live. Disease burden due to acute respiratory infection is 10–50 times higher in developing countries when compared to developed countries. The aim of this study was to assess risk factors of acute respiratory infection among under-five children attending Public hospitals in Southern Tigray, Ethiopia 2016/2017.

**Methods:**

Institution based case control study was conducted from Nov 2016 to June 2017. Interviewer administered structured questionnaire was used to collect data from a sample of 288 (96 cases and 192 controls) children under 5 years of age. Systematic random sampling was used to recruit study subjects and SPSS version 20 was used to analyze the data. Bivariate and multivariate analysis were employed to examine statistical association between the outcome variable and selected independent variables at 95% confidence level. Level of statistical Significance was declared at *p* < 0.05. Tables, figures and texts were used to present data.

**Result:**

One hundred sixty (55.6%) and 128 (44.4%) of the participants were males and females respectively. Malnutrition (AOR = 2.89; 95%CI: 1.584–8.951; *p* = 0.039), cow dung use (AOR =2.21; 95%CI: 1.121–9.373; *p* = 0.014), presence of smoker in the family (AOR = 0.638; 95% CI: 0.046–0.980; *p* = 0.042) and maternal literacy (AOR = 3.098; 95%CI: 1.387–18.729; *p* = 0.021) were found to be significant predictors of acute respiratory infection among under five children.

**Conclusion:**

According to this study maternal literacy, smoking, cow dung use and nutritional status were strongly associated with increased risk of childhood acute respiratory infection. Health care providers should work jointly with the general public, so that scientific knowledge and guidelines for adopting particular preventive measures for acute respiratory infection are disseminated.

## Background

Acute Respiratory infection (ARI) accounts for an average 94,037000 disability adjusted life years (DALY) and 1.9 million mortalities throughout the world. The disease is among the most common causes of both illness and mortality in children aged below 5 years [1, 2]. Acute respiratory infection contributes 2 to 4% of deaths in children less than 5 years of age in developed countries. These causes contribute 19 to 21% of child death in the eastern Mediterranean, Africa and South East Asia regions [3]. Although the frequency of ARI is similar in both the developed and developing countries, mortality due to ARI is 10–50 times higher in developing countries [4].

In countries with high pediatric population, one fourth of all pediatric hospital admissions are mainly due to ARI. Each year, 3% of all children less than 12 months of age need to be admitted for moderate or severe lower respiratory tract infections [5].

Ethiopia has made investments to reduce the morbidly and mortality of ARI. Integrated management of common childhood illness and community case management are among the programme initiatives scaled up nationally to address ARI in the country [6].

There are many socio-cultural, demographic and environmental risk factors that predispose children less than 5 years to acquire Respiratory Tract Infections (RTIs). Even though many of these risk factors are preventable [7], they have not been documented in many regions in Ethiopia making it difficult to develop algorithms for the management of this group of patients.

Considering the feasibility of the study design and the dynamic nature of the pediatric population a case control study design was employed aimed at determining the associated risk factors of ARI amongst children under 5 years of age who attend the southern Tigray Public Hospitals.

## Methods

### Study design

Since the pediatric population is a dynamic population and difficult to follow-up, an institutional based unmatched case control study design was employed to collect data on under five children’s risk factors of acute respiratory infection.

### Source population and study population

The source population was all children less than 5 years of age in Southern zone of Tigray coming to public Hospitals. The study population was all sampled children of less than 5 years of age attending in the five public Hospitals during the data collection period.

### Eligibility criteria

Children of under 5 years of age who diagnosed with ARI at time of data collection period in which their mothers accept to provide informed consent for their children. Exclusion criteria were children whose mothers or care takers were refused to participate in the study.

### Selection of cases

The data collectors identified children who were diagnosed with ARI by the physician in the outpatient clinic. The data collectors then selected the study subjects by systematic random sampling method (an interval of 2 was used to get the actual study participants). Following this selection, after spoken informed consent was given participants were included in the study.

### Selection of controls

The study data collectors selected the controls on meeting the definition of controls. The recruitment of the controls was done as for the cases as outlined in the above procedure.

### Study variables

Dependent variable was acute respiratory infection. Independent variables were, Parental Social Demographic factors, Child Physiological/nutritional factors and Environmental characteristics.

### Conceptual framework

**Fig. 1:**
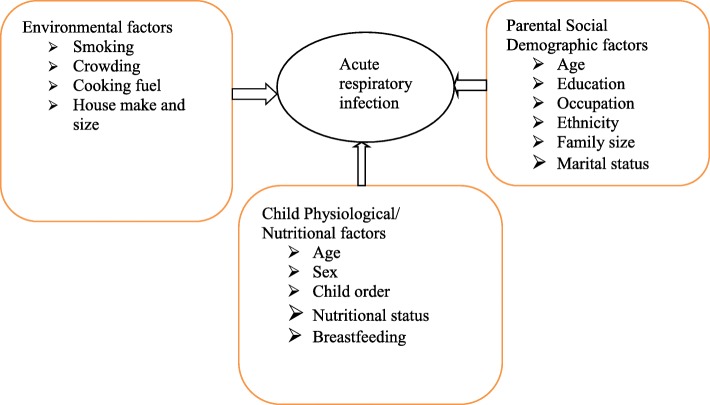
conceptual framework to assess risk factors of acute respiratory infection among under-five children

The conceptual frame work of this study illustrates acute respiratory infection and its risk factors. As depicted in Fig. 1, conceptual framework is developed for this research after reviewing the relevant literatures (Fig. 1).

### Sample size determination

Sample size was calculated using Epi Info 7.0 StatCalc program by taking assumptions of 95% confidence level, two controls for each case, 80% power and 18.3% controls having wasting syndrome giving OR of 2.42 [8], Giving a total sample of 261 (87 cases and 174 controls). Adding 10% non-response rate the final sample was found to be 288 (96 cases and 192 controls). Wasting is selected because it was the exposure variable that gave the highest sample size for cases and controls among the other variables in a study conducted in Kenya [8].

### Sampling procedure

**Fig. 2:**
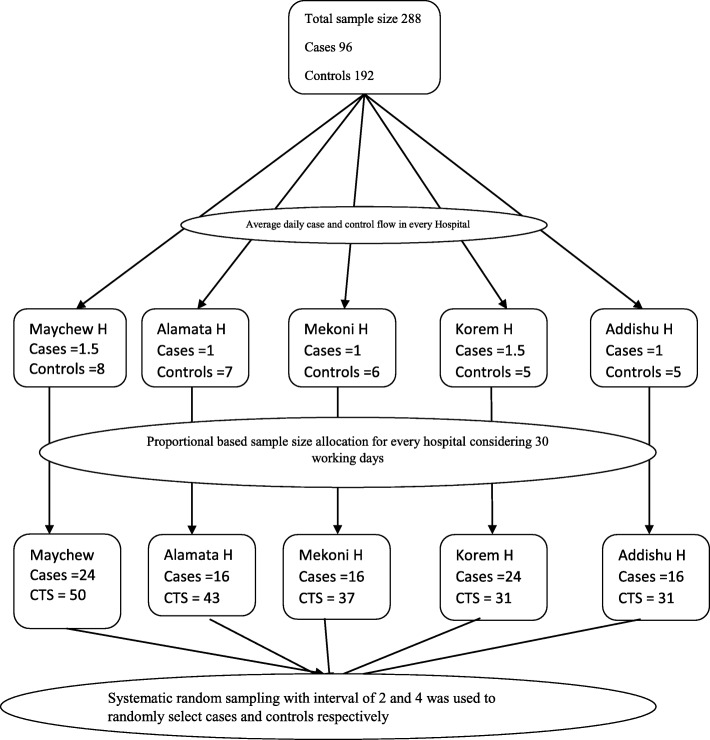
schematic presentation of sampling procedure of a research project

All the five public hospitals in the zone were included in the study. As a marker for proportional sample size allocation for the hospitals, client flow of three consecutive previous months prior to the data collection period was observed. Systematic random sampling was used to recruit study subjects (Fig. 2).

### Data collection tools

Interviewer administered structured questionnaire was used to collect data on risk factors of acute respiratory infection among under five children attending the five public hospitals. The questionnaire was adopted from previous studies and modified accordingly; it was first developed in English and translated in to the local Tigrigna language, and was then translated back to English to check the consistency. The data collection tool is included as an Additional file 1.

### Data collection process

Seven individuals who have completed their BSc in nursing from a recognized University were recruited (five of them for data collection and two of them for supervision) and each hospital’s chief executive officer met and asked for permission. The data collection was held for a total of 8 months from November 2016 - June/2017.

### Operational definition

Acute Respiratory Infections (ARI) in children: children with any one or combination of symptoms and signs like cough, sore throat, rapid breathing, noisy breathing, chest in drawing, at any time in the last 2 weeks.

### Cases

Children less than 5 years of age diagnosed with ARI in the hospitals and those referred from other health facilities with the diagnosis of ARI.

### Controls

Children who visit the hospitals for diagnosis other than ARI.

### Wasting

Refers to low weight-for-height where a child is thin for his/her height but not necessarily short.

### Data quality control and assurance management

The data collectors were trained for 1 day and the supervisors were visiting the data collectors once a day to check if they collect the data appropriately. Pretest was carried out on 10% of the sample in two health centers of the zone which were not included in the actual data collection 2 weeks before the actual data collection and the questions were revised based on the response obtained so that questions that create ambiguity were rephrased.

### Data analysis procedure

The data was first recorded and cleaned then analyzed using SPSS version 20 software statistical packages. Missing values were treated by SPSS too. Frequency and proportions were used to describe the study population in relation to relevant variables. Binary logistic regression was computed to assess statistical association via Odds ratio, and significance of statistical association was assured or tested using 95% confidence interval and *P*-value (0.05). Bivariate and multivariate analysis was employed to examine the relationship or statistical association between the outcome variable and selected independent variables. Variables which were significant at *p* < 0.05 in the bivariate analysis were taken to multivariate analysis to control the possible confounders. Results were presented using tables, figures and texts.

### Ethical consideration

Ethical clearance was secured from Mekelle University College of health science IRB (research committee).

## Result

### Socio demographic characteristics of the respondents

A total of 288 (96 cases and 192 controls) under five children were included in the study with a response rate of 100%. The children were aged between 4 and 59 months with median age of 16.5 months (Mean ± SD; 20.8 ± 13.9).

Fifty seven (62%) of the cases and 100 (51%) of the controls were rural dwellers. About three fourth of the respondents 227(78.8%) were Orthodox in religion. Thirty six (39.1%) of the mothers of cases and 48(24.5%) of mothers of controls were illiterate with only 4(2%) of mothers of controls completed college program. Fifty two (56.5%) of the cases and 108(55.1%) of the controls were males (Table 1).

**Table 1:** Association of child and parental characteristics with acute respiratory infections among under-five children in Southern Tigray public hospitals, Ethiopia, 2016/2017 (cases 96, controls 192)

Variables	Participant type		COR (95% CI)
	Cases	Controls	
Age of mother	n(%)	n(%)	
< 20	6 (6.5)	14 (7.1)	1
20–25	25 (27.2)	59 (30.1)	1.011 (0.349–2.933)
26–30	24 (26.1)	49 (25)	0.875 (0.299–2.561)
31–35	16 (17.4)	43 (21.9)	1.152 (0.378–3.514)
36–40	17 (18.5)	25 (12.8)	0.630 (0.202–1.966)
> 40	4 (4.3)	6 (3.1)	0.643 (0.132–3.140)
Residence			
Urban	38 (39.5)	93 (48.4)	1
Rural	58 (60.5)	99 (51.6)	0.640 (0.386–1.060)
Religion			
Orthodox	74 (80.4)	153 (78.1)	1
Muslim	15 (16.3)	41 (20.9)	1.322 (0.688–2.541)
Protestant	2 (2.2)	2 (1)	0.484 (0.067–3.501)
Educational status of mother			
Unable to read and write	36 (39.1)	48 (24.5)	1.211 (0.576–3.033)
Read and write	19 (20.7)	42 (21.4)	1.658 (0.829–3.316)
Primary	16 (17.4)	28 (14.3)	1.312 (0.619–2.781)
Secondary	15 (16.3)	59 (30.1)	2.950 (1.446–6.017)
Preparatory	6 (6.5)	15 (7.7)	1.875 (0.662–5.309)
Occupation of mother			
Government employee	9 (9.8)	32 (16.3)	1
Student	1 (1.1)	4 (2)	1.125 (0.111–11.365)
Farmer	16 (17.4)	50 (25.5)	0.879 (0.347–2.226)
Merchant	13 (14.1)	32 (16.3)	0.692 (0.260–1.847)
Daily worker	12 (13)	7 (3.6)	0.164 (0.050–0.539)
House wife	41 (44.6)	71 (36.2)	0.487 (0.212–1.121)
Monthly income (ETB)			
< 1000	23 (25)	45 (23)	1.304 (0.673–2.526)
1000–2500	40 (43.5)	77 (39.3)	0.984 (0.523–1.849)
2500 and above	29 (31.5)	74 (34.8)	1
Household family size			
4 or less	27 (29.3)	76 (38.8)	1
5–7	47 (51.1)	108 (55.1)	0.816 (0.468–1.425)
8 and above	18 (19.6)	12 (6.1)	0.237 (0.101–0.555)
Child age (months)	n(%)	n(%)	
< 6	1 (1.1)	10 (5.1)	1
6–12	35 (38)	74 (34.8)	0.211 (0.26–1.717)
13–24	24 (26.1)	55 (28.1)	0.229 (0.28–1.892)
25–59	32 (34.8)	57 (91.1)	0.178 (0.22–1.456)
Child sex			
Male	55 (57.2)	105 (54.6)	1
Female	41 (42.8)	87 (43.4)	1.059 (0.643–1.745)
Place where child delivered			
Hospital	37 (40.2)	99 (50.5)	1
Health center	47 (41.1)	87 (44.4)	0.692 (0.412–1.162)
Home	8 (8.7)	10 (5.1)	0.467 (0.171–1.274)
Number of siblings			
0	15 (16.3)	48 (24.5)	1
1–2	23 (25)	64 (32.7)	0.870 (0.411–1.842)
3 and above	54 (58.7)	84 (42.9)	0.486 (0.248–0.953)
Number of under five children			
1	75 (81.5)	171 (87.2)	1
2	17 (18.5)	25 (12.8)	0.645 (0.329–1.265)
Birth order			
First	13 (14.1)	50 (25.5)	1
Second	14 (15.2)	27 (13.8)	0.501 (0.206–1.229)
Third	13 (14.1)	38 (19.4)	0.760 (0.316–1.827)
4th and above	52 (56.5)	81 (41.3)	0.405 (0.201–0.808)
Period of breast feeding			
Not breast fed	2 (2.2)	5 (2.6)	1
Less than 4 months	1 (1.1)	4 (2)	1.600 (0.104–24.703)
4–6 months	2 (2.2)	3 (1.5)	0.600 (0.053–6.795)
6 months and above	36 (39.1)	55 (28.1)	0.611 (0.112–3.321)
Continuing	51 (55.4)	129 (65.8)	1.012 (0.190–5.383)
Nutritional status			
Wasted	7 (7.6)	20 (11.2)	1.51 (1.779–9.296)
Not wasted	84 (92.4)	157 (88.8)	1

*OR* Odds ratio, *95% CI* 95% confidence interval, *p* Level of significance using chi-square test, *p* < 0.05 was considered significant

### Factors associated with acute respiratory infection

#### Child and parent related factors

Among variables under this category maternal literacy, maternal occupation and household family size demonstrate significant association with acute respiratory infection of under five children at the bivariate analysis.

Most of the respondents were illiterate with 36 (39.1%) of caretakers of cases being unable to read and write and 59(30%) caretakers of controls having at least secondary education. A significant association was found between maternal literacy and risk of ARI by bivariate analysis (COR = 2.95, 95% CI: 1.446–6.017; *p* = 0.04).

As shown in Table 1, over 50% of the homes had between 5 and 7 persons living in the house. A significant association was found between family size and risk of ARI by bivariate analysis (OR = 0.237 (0.101–0.555, *p* = 0.02) (Table 1).

Number of siblings, birth order and nutritional status were found to show significant association with under five children acute respiratory infection in the bivariate analysis.

The highest proportion of children had 3 and above siblings, among them were 54 (58.7%) cases and 84 (42.9%) control children. Number of siblings were found to be significantly associated with ARI (*p* = 0.041). Birth order of the child were found to be significantly associated with risk of ARI (*p* = 0.048).

Overall, malnutrition (severe and moderate; MUAC< 12.5 mm) was found significantly associated with increased risk of ARI (COR = 1.51, 95% CI: 1.779–9.296; *p* = 0.001) in the bivariate analysis (Table 1).

#### Environmental factors

Among variables of this category cow dung use and presence of smoker in the house illustrate significant association with acute respiratory infection of under five children in the bivariate analysis.

Among the fuel types used cow dung for cooking was found to be associated with Acute respiratory infection on bivariate analysis (*p* = 0.002). A significant association was found between smoking and risk of ARI by Bivariate analysis (OR = 0.139, 95% CI: 0.043–0.444) (Table 2).

**Table 2:** Association of environmental characteristics with acute respiratory infections among under five children in southern Tigray public hospitals, Ethiopia 2016/2017 (cases 96, controls 192)

Variables	Participant		COR (95% CI)
	Cases	Controls	
House type	n (%)	n (%)	
Mud	58 (63)	131 (66.8)	0.295 (0.48–1814)
Stone and bricks	31 (33.7)	63 (32.1)	0.900 (0.530–1.528)
Iron sheet	3 (3.3)	2 (1)	1
Kitchen place			
Inside house	30 (32.6)	70 (35.7)	1.148 (0.679–1940)
Outside house	62 (67.4)	126 (64.3)	1
Kitchen have chimney			
Yes	50 (54.3)	90 (45.9)	1
No	42 (45.7)	106 (54.1)	1.402 (0.853–2.305)
Child carried on the back while cooking			
Always	6 (6.5)	16 (8).2	0.858 (0.320–2.302)
Sometimes	27 (29.3)	45 (23)	0.625 (0.218–1.791)
Never	59 (64.1)	135 (68.9)	1
Time stay in kitchen			
One hour	39 (42.4)	95 (48.5)	1
Two hour	48 (52.2)	91 (49.5)	0.830 (0.499–1.379)
Three hour	5 (5.4)	4 (2)	0.328 (0.084–1.288)
Fuel type			
Wood			
Yes	87 (84.6)	174 (88.8)	0.456 (0.166–1241)
No	5 (5.4)	22 (11.2)	1
Cow dung			
Yes	45 (49.9)	110 (56.1)	1.334 (1.001–4.973)
No	47 (58.1)	86 (44.9)	1
Charcoal			
Yes	36 (39.1)	104 (53.1)	1.758 (1.062–2.911)
No	56 (60.9)	92 (46.9)	1
Wood and cow dung			
Yes	38 (41.3)	58 (29.6)	0.597 (0.356–1.001)
No	54 (58.7)	138 (70.4)	1
Wood and charcoal			
Yes	33 (35.9)	93 (47.4)	1.164 (0.969–2.688)
No	59 (64.1)	103 (52.6)	1
Smoker in the house			
Yes	12 (13)	4 (2)	0.139 (0.043–0.444)
No	80 (87)	192 (98)	1

*OR* Odds ratio, *95% CI* 95% confidence interval, *p* Level of significance using chi-square test, *p* < 0.05 was considered significant

#### Overall factors of acute respiratory infection in children

In the bi-variable logistic regression analysis, variables such as maternal literacy, maternal occupation, family size, birth order, number of siblings, presence of smoker in the house, cow dung use and wasting were appeared to be associated with acute respiratory infection. Those variables which were significant in bivariate analysis at *p* < 0.05 were taken to multivariate analysis to control the possible confounders. Then on multivariate analysis only maternal literacy, cow dung use and nutritional status were found to be associated with ARI.

Children from houses which used cow dung for their fuel were 2 times (AOR =2.21; 95%CI: 1.121–9.373; *p* = 0.014) more likely to develop ARI. Similarly, ARI was about 3 times (AOR = 2.89; 95%CI: 1.584–8.951; *p* = 0.039) more common among under five children who were wasted (Table 3).

**Table 3:** Overall factors associated with acute respiratory infection among under-five children in Southern Tigray public hospitals, Ethiopia, 2016/2017 (multivariate analysis).(cases 96, controls 192)

Variables	Participant type		AOR (95% CI)
	Cases	Controls	
Educational status of mother			
Unable to read and write	36	48	1.987 (0.354–7.768)
Read and write	19	42	2.249 (0.803–6.298)
Primary	16	28	1.656 (0.446–6.143)
Secondary	15	59	3.098 (1.387–18.729)^*^
Preparatory	6	15	2.012 (0.274–14.771)
College/University	0	4	1
Occupation of mother			
Government employee	9	32	1
Student	1	4	0.052 (0.002–1.258)
Farmer	16	50	3.134 (0.537–18.288)
Merchant	13	32	0.888 (0.165 (4.777)
Daily worker	12	7	0.206 (0.032–1.350)
House wife	41	71	1.412 (0.291–6.861)
Household family size			
4 or less	27	76	1
5–7	47	108	1.635 (0.279–9.586)
8 and above	18	12	0.627 (0.079–4.978)
Cow dung use			
Yes	45	110	2.21 (1.121–9.373)^*^
No	47	86	1
Smoker in the house			
Yes	12	4	0.638 (0.046–0.1.01)
No	80	192	1
Nutritional status			
Wasted	7	20	2.89 (1.584–8.951)^*^
Not wasted	84	157	1
Number of siblings			
0	15	48	1
1–2	23	64	4.186 (0.590–29.675)
3 and above	54	84	2.766 (0.272–28.183)
Birth order			
First	13	50	1
Second	14	27	0.182 (0.025–1.307)
Third	13	38	0.130 (0.015–1.157)
4th and above	52	81	0.229 (0.037–1.411)

Hosmer and lemeshow’s goodness of model test was found to be chi-square of 13.997 with *p*-value of 0.82 which implies the goodness of the model to predict the outcome

^*^Significant at *p* < 0.05

## Discussion

This study found a significant association of malnutrition with ARI. The result contrasts to a case control study conducted in Kenya which reports an inverse relationship between ARI and wasting (OR = 2.42) [8]. Findings of this study also compared with case control study conducted in Zimbabwe which reported that current and past malnutrition were associated with ARI in children under five with OR = 2.67 [9]. Earlier study conducted in Riyadh city also reported that ARI was more seen in undernourished children (22.2%vs 15.8%; *p* = 0.001) with increased incidence of ARI due to weakening nutritional status (*P* = 0.05) [8]. Declining MUAC (*p* = 0.001) was reported to be associated with ARI and in the nonappearance of other factors malnutrition alone significantly affect the ARI in under 2 years children [10]. One possible explanation for this contrasting finding might be that the effect of lessened cellular immunity in undernourished children which makes them more disposed to ARI. Acute Respiratory Infections usually occur more often, last longer, and are starker in malnourished children, classically because the mucous membranes and other mechanical structures designed to keep the respiratory tract clear are impaired, and the immune system has not developed properly [11].

This study also found a noteworthy association of maternal literacy with ARI but not with father’s literacy. Parker RL [12], revealed risk of ARI declined with education of parents. This might be because usually father remains outside for job most of the times but mother is always in the home taking care of children and household activities. Mother due to her close connotation with child knows the minor variations in child’s health than father. Due to such factors mother’s educational status might play important role in child’s disease than father’s literacy.

Cow dung use was the other variable found to be associated with ARI in this study. This result is in agreement with study done by Vinod Mishra et al. [12], who revealed an association of cow dung use with ARI (OR = 2.2). This could be because of the high daily concentrations of pollutants found in such settings and the large amount of time young children spend with their mothers doing household cooking.

## Limitations of the study

- Diagnosis of ARI was based on clinical WHO IMNCI classification guideline, which could introduce misclassification bias which could lead to selection bias.
- Being institution based case control the study may have limitation in the generalizability of the findings.
- Also, this study selectively addressed certain factors of under-five ARI while various factors are found to cause the diseases

## Conclusion

This study revealed that, maternal literacy, cow dung use, and nutritional status were strongly associated with increased risk of childhood ARI.

Based on the findings in this study, the following are recommended.

- Each Wereda’s Health Office of the zone, in teamwork with the health services in the wereda, ought prepare plans to implement community-based interventions focused towards better food, supplementation (vitamin supplements or fortified milk) to have significant optimistic benefits in dropping malnutrition
- Health care providers in partnership with other participants should have plan to provide health education and choices of cooking other than cow dung.
- Investigators should conduct extra studies related to this problematic in the area so that all the likely factors could be explored
- The FMOH should give weight to mark the mothers familiar concerning their health and kids’ health as when design to control childhood diseases

Generally, it is suggested that the policy makers and academicians/health care providers should effort together to make a communication stage with the general community, through which scientific knowledge and guidelines for adopting particular preventive measures for ARI are disseminated. Since community responses to the ARI epidemic are dynamic, continual surveillance of community responses is valuable and would facilitate relevant governmental risk communication and health education efforts.

## Supplementary information

**Additional file 1:** Questionnaire to assess risk factors of acute respiratory tract infections among under five children attending public hospitals in southern Tigray, Ethiopia.

## Data Availability

The datasets used and/or analysed during the current study are available from the corresponding author on reasonable request.

## Abbreviations

AIDS: Acquired Immune Deficiency Syndrome
AOR: Adjusted Odds Ratio
ARI: Acute respiratory infection
CI: Confidence interval
COR: Crude Odds Ratio
DALY’s: Disability adjusted life years
GBD: Global Buren of Disease
IMCI: Integrated Management of childhood Illness
LRTI: Lower respiratory tract infection
MOH: Ministry Of Health
OR: Odds ratio
PHC: Primary Health Care
RSV: Respiratory Synctial Virus
SARI: Severe Acute Respiratory Infections
UNICEF: United Nations Children’s Fund
URTI: Upper Respiratory Tract Infection
USA: United States of America
WHO: World Health Organization
