# Supplementary material for: Risk factors of acute respiratory infections among under five children attending public hospitals in southern Tigray, Ethiopia, 2016/2017
Source: BMC Pediatr. 2019 Oct 25;19:380. doi: 10.1186/s12887-019-1767-1 (PMC6814116; doi:10.1186/s12887-019-1767-1)
Supplement: Supplementary file 1 — Additional file 1: Questionnaire to assess risk factors of acute respiratory tract infections among under five children attending public hospitals in southern Tigray, Ethiopia. [file 12887_2019_1767_MOESM1_ESM.docx]

**Additional file 1**

**Participant's information sheet**

Mekelle University College of health science, department of nursing, individual information sheet form for the study on risk factors of acute respiratory infection among under five children attending public hospitals in Southern Tigray, Ethiopia.

You are kindly invited to participate in this study, which involves all under five years children who are visiting public hospitals of southern Tigray. The aim of this study is to assess risk factors of acute respiratory infection among under-five children attending Public hospitals in Southern Tigray. Assessing acute respiratory infection among under five children is very crucial and is the corner stone in managing the problem and preventing complications. Therefore this study will important in creating awareness among health professionals by giving evidence about under five acute respiratory infection.

**A. Purpose:** The purpose of this study is to assess risk factors of acute respiratory infection among under-five children attending Public hospitals in Southern Tigray.

**B. Duration:** The duration of this study is from Nov 2016 to June 2017

**C. Procedures to be carried on:** The procedure of data collection is easy and straight forward; data concerning your socio demographic characteristics, environmental characteristics and nutritional status using standardized questioner interviewers (nurses).

**D. Risk and discomfort:** There will no any risk associated during data collection

**E. Expected benefits**: You will not get special and direct benefit from this study. However, the finding of this survey will be useful for all under five children in the future because this study result will be able us to understand the risk factors of acute respiratory infection which is useful in delivering improved health service based on the causes.

**F. Confidentiality:** All your personal information collected for the purpose of the present study will be kept confidential.

**G. Compensation:** No compensation will be provided by participating in this study.

**H. Termination of the study:** Participation in the study is voluntary, and refusal to participate involves no penalty or loss of benefits to which you are otherwise entitled. The study participants have a right to Keep hold information; decline to cooperate in the study, to refuse provision of data. I would also like to inform you that this study will be approved by Mekelle University college of health sciences ethical Review Committee (IRB) and approved by department of nursing. If you have any question about the right of the study participant the address is:

College of health Science, Mekelle University

Office of research and community services

P.O. Box 1871. Mekelle, Ethiopia

Tel. 251-914703261

If you have question about the study the address of the principal investigator is:

**SIELU ALEMAYEHU DESTA**

Department of Nursing

College of Health Science, Mekelle University

P.O. Box 1871, Mekelle, Ethiopia

Tel: 0963480274, e mail address: [siealem@yahoo.com](mailto:Kalushaibex@yahoo.com)

Advisor**:** Kalayou Kidanu (BSc, MSc Assistant Prof.)

Department of Nursing, College of Health Science, Mekelle University

Mobile: +251 912 11 77 19

**Informed consent**

I (the respondent), the undersigned, am told that the investigator is going to conduct study in this zone to determine the risk factors of under five Acute respiratory infection and he acquainted with me the first time s/he meets. I am also informed that the result of the study will be used by both the zone and the hospital health office to commence appropriate strategies to battle this problem. I am, too, told that the research will benefit the community in general including me, the respondent, and that the research will not inflict any harm to me. I have been told that I have full right to have enough time to understand and then take part in the study on the basis of my interest and Besides, I am briefed that I will be interviewed for not more than 20 minutes. Moreover, I am notified that my participation in the study is entirely voluntarily, and that I can quit from the study any time I want. Likewise, I am enlightened that I will not be subjected to any form of punishment following my failure to participate in the study. In the same way, I am explained that the information collected from me will not by any means be disclosed to any people other than those participating in the study unless obtained permission from me. Equally, I am told that I can ask them questions I found difficult or any type otherwise.

Signature_______________________

Date______________________

**English version Questionnaire**

**Case Control**

| **Parental Socio-demographic characteristics** | | | |
| --- | --- | --- | --- |
| Q.No | Question | Response and Code | Comments |
|  | Identity |  |  |
|  | Age (caretaker) | …………….. |  |
|  | Residence | 1. Urban 2. Rural |  |
|  | Ethnicity | 1. Tigray 2. Amhara 3. Afar 4. Other (specify)___________________ |  |
|  | Religion | 1. Orthodox 2. Muslim 3. Protestant 4. Catholic 5. Other specify_______________________ |  |
|  | Occupational status of the mother | 1. Government 2. Private 3. Farmer 4. Trader 5. Daily wage labourer 6. Other (specify)…. |  |
|  | Occupation status of  the father | 1. Government 2. Private 3. Farmer 4. Trader 5. Daily wage labourer 6. Other (specify)…. |  |
|  | Education status of  the mother | 1. Illiterate 2. Primary level 3. Upper primary level 4. High school level 5. Higher Secondary 6. Graduate 7. Others (specify) |  |
|  | Education status of  The father | 1. Illiterate 2. Primary level 3. Upper primary level 4. High school level 5. Higher Secondary 6. Graduate 7. Others (specify) |  |
| **Information on family members** | | | |
|  | Type of family | 1. Nuclear family 2. Joint family |  |
|  | Total number of  family members | 1. 2 2. 3 3. 4 4. 5 5. 6 6. Others (specify)… |  |
|  | Number of siblings | 1. 0  2. 1-2  3. 3 and above |  |
|  | Number of under  five children in the  house hold | 1. 1 2. 2 3. 3 4. Other …. |  |
|  | How many arc  sharing the child's  bed room | 1. 1 2. 2 3. 3 4. >3 5. Others specify……… |  |
|  | Is there any smoker in the house hold? | 1. Yes 2. No 3. Don't know |  |
|  | If yes, do they smoke inside the house? | 1. Inside 2. Outside |  |
| **Environmental characteristics** | | | |
| 18 | Location of house in terms of road access | 1. Next to main road 2. Not next to main road |  |
| 19 | Type of house | 1. Mud 2. Stone and bricks 3. Iron sheet and Timber |  |
| 20 | Type of floor | 1. Mud 2. Cow dung 3. Cement 4. Others specify |  |
| 22 | What was your  household  expenditure last  Month? | ETB…… |  |
| 23 | Where is the location of kitchen? | 1. Inside 2. Outside |  |
| 24 | Does the kitchen have a chimney? | 1. Yes 2. No |  |
| 25 | Fuel used and  location of stoves | \| Fuel \| Presence \| Location \| \| --- \| --- \| --- \| \| Wood \| 1. Yes 2. No \| 1. Inside House  2. Outside House \| \| Cow dung \| 1. Yes 2. No \| 1. Inside House  2. Outside House \| \| Charcoal \| 1. Yes 2. No \| 1. Inside House  2. Outside House \| \| Kerosene \| 1. Yes 2. No \| 1. Inside House  2. Outside House \| \| Electricity \| 1. Yes 2. No \| 1. Inside House  2. Outside House \| |  |
| 26 | Does the kitchen have windows? | 1. Yes 2. No |  |
| 27 | How often do you carry the child while cooking? | 1. Always  2. Sometimes  3. Never |  |
| 28 | Time spent in the  Kitchen | 1. 1 hr 2. 2 hrs 3. 3 hrsand above |  |
| **Child physiological and immunological factors** | | | |
| 29 | Age of the child | (in months)___________ |  |
| 30 | Sex of the child | 1. Male 2. Female |  |
| 31 | Where did you go  for ante-natal check  up | 1. Government Hospital 2. Health centre 3. Private Hospital 4. Private Clinic 5. Others specify |  |
| 32 | Where did you give birth to your last child? | 1. Government Hospital 2. Health center 3. Private Hospital 4. Home 5. Others specify |  |
| 33 | Mother's age at  Delivery | 1. I 8 yrs 2. 2 0 yrs 3. 25-30 yrs 4. >30 yrs |  |
| 34 | Did you breast feed the baby within an hour after delivery? | 1. Yes 2. No |  |
| 35 | How long did you breast feed your child? | 1. Not breast fed 2. Less than 4 months 3. 4-6 months 4. 6months and above 5. Continuing *(tick this if the child is*   *still breastfeeding)* |  |
| 36 | What is the Birth order of the child in the family? | 1. 1 2. 2 3. 3 4. Other ………… |  |
| 37 | Who is the primary care taker of your child/baby? | 1. Mother 2. Grandmother 3. Others specify….. |  |
| 38 | Did the child had fever at any time in the last 2 weeks? | 1. Yes 2. No 3. Don't know |  |
| **Child nutritional status** | | | |
| 42 | MUAC | (In cm)………. |  |

**Screening for malnutrition using Mid-Upper Arm Circumference**

**Materials**

1. MUAC 4 colour Tape

**Steps for taking the MUAC measurement of a child**

1. Determine the mid-point between the elbow and the shoulder (acromion and olecranon) as shown on the picture below.

2. Place the tape measure around the LEFT arm (the arm should be relaxed and hang down the side of the body).

3. Measure the MUAC while ensuring that the tape neither pinches the arm nor is left loose.

4. Read the measurement from the window of the tape or from the tape.

5. Record the MUAC to the nearest 0.1 cm or 1mm in the interview form.

**Interpretation**:

Using a 4-colour tape:

• Measurement in the green zone means the child is properly nourished (>13.5cm);

• Measurement in the yellow zone means that the child is at risk of malnutrition (Between 12.5 – 13.5);

• Measurement in the orange zone means that the child is moderately malnourished (Between 11-12.5cm);

• Measurement in the red zone means that the child is severely malnourished (<11cm).

**Note**: Repeat the measurement two times to ensure an accurate interpretation.
